# Protective Effect of *Portulaca oleracea* on Streptozotocin-Induced Type I Diabetes-Associated Reproductive System Dysfunction and Inflammation

**DOI:** 10.3390/molecules27186075

**Published:** 2022-09-17

**Authors:** Hassan Rakhshandeh, Hamed Rajabi Khasevan, Anella Saviano, Mohammad Reza Mahdinezhad, Vafa Baradaran Rahimi, Sajjad Ehtiati, Leila Etemad, Alireza Ebrahimzadeh-bideskan, Francesco Maione, Vahid Reza Askari

**Affiliations:** 1 Pharmacological Research Center of Medicinal Plants, Mashhad University of Medical Sciences, Mashhad 9177948564, Iran; rakhshandehh@mums.ac.ir (H.R.); rajabih961@mums.ac.ir (H.R.K.); baradaranrv@mums.ac.ir (S.E.); 2 ImmunoPharmaLab, Department of Pharmacy, School of Medicine and Surgery, University of Naples Federico II, Via Domenico Montesano 49, 80131 Naples, Italy; anella.saviano@unina.it; 3 Department of Clinical Biochemistry, Faculty of Medicine, Mashhad University of Medical Sciences, Mashhad 9177948564, Iran; mahdinezhadmr961@mums.ac.ir; 4 Department of Cardiovascular Diseases, Faculty of Medicine, Mashhad University of Medical Sciences, Mashhad 9177948564, Iran; ehtiatis961@mums.ac.ir; 5 Pharmaceutical Research Center, Pharmaceutical Technology Institute, Mashhad University of Medical Sciences, Mashhad 9177948564, Iran; etemadl@mums.ac.ir; 6 Department of Drug Control, School of Pharmacy, Mashhad University of Medical Sciences, Mashhad 9177948564, Iran; 7 Department of Anatomy and Cell Biology, Faculty of Medicine, Mashhad University of Medical Sciences, Pardis Campus, Azadi Square, Mashhad 9177948564, Iran; ebrahimzadehba@mums.ac.ir; 8 Applied Biomedical Research Center, Mashhad University of Medical Sciences, Mashhad 9177948564, Iran; 9 Department of Pharmaceutical Sciences in Persian Medicine, School of Persian and Complementary Medicine, Mashhad University of Medical Sciences, Mashhad 9177948564, Iran; 10 International UNESCO Center for Health-Related Basic Sciences and Human Nutrition, Mashhad University of Medical Sciences, Mashhad 9177948564, Iran

**Keywords:** diabetes mellitus, infertility, inflammation, oxidative stress, *Portulaca oleracea*

## Abstract

Background: Type-one diabetes (T1D), a chronic autoimmune disease with marked inflammatory responses, is associated with infertility complications and implications. Based on the anti-diabetic, antioxidant, and anti-hyperlipidemic potential of *Portulaca oleracea* (PO), this study aimed to evaluate the protective effect of this plant extract on streptozotocin-induced type-I-diabetes-associated reproductive system dysfunction and inflammation. Methods: Male rats were randomly divided into four experimental groups: control, diabetic, and treatment/s (PO extract at 100 or 300 mg/kg/daily). Then food and water consumption, body, testis and epididymis weights, histopathological evaluation, seminiferous tubules diameter, sperm count and motility, glucose levels, sex hormones, and inflammatory and oxidative stress markers were evaluated. Results: Our results showed that streptozotocin-induced diabetes significantly increased food and water consumption; increased glucose, MDA, TGF-β1, and TNF-α levels; and decreased the seminiferous tubules diameter, sperm count and motility, levels of LH, testosterone, total thiol, VEGF, and SOD activity. Interestingly, PO extract (phytochemically characterized by using liquid chromatography–mass spectrometry to detect bioactive molecules) significantly ameliorated these parameters and histopathological indexes’ damage in rats. Conclusion. Even if more preclinical assessments are needed to better characterize the mechanism/s of action, the results of this study will pave the way for the rational use of PO on diabetic-associated clinical complications and implications.

## 1. Introduction

Diabetes mellitus (DM) is considered one of the most critical and fast-increasing prevalence health concerns worldwide [1]. The prevalence of diabetes, according to the international diabetes federation, was 463 million in 2016, and it is predicted to reach 578 million patients in 2030 and 700 million patients in 2045 [2]. Type-one DM, a chronic autoimmune disease associated with marked inflammatory responses, is characterized by insulin deficiency due to loss of the insulin-producing β cells of the pancreatic Langerhans islets with a consequent disorder in glucose metabolism [3]. In addition, DM is related to the long-term severe damage and failures to various organs that cause complications, including retinopathy, nephropathy, peripheral neuropathy, cardiovascular disorders, and sexual dysfunction [4]. The disturbance of the male reproductive system and infertility is one of the most important and concerning complications of DM. Several animal and human studies have emphasized the detrimental effects of DM on sexual functions and parameters [5,6].

*Portulaca oleracea* L. (PO), commonly called purslane or hogweed, is an annual grassy plant belonging to *Portulacaceous* that has been widely used as a potherb in Central European, Mediterranean, and Asian countries [7,8,9,10,11]. PO possess numerous active components, including flavonoids such as kaempferol and apigenin; terpenoids such as Portuloside A and B; organic acids such as α-linolenic acid and palmitic acids; and minerals and vitamins [12]. In addition, several pharmacological activities have been reported for PO, including antioxidant, anti-inflammatory, anti-cancer, immune-modulating, and analgesic properties [7,8,9,10,11,12,13]. Additionally, it has been demonstrated that PO exerts anti-diabetic, glucose-lowering, and insulin-elevating effects in preclinical settings and investigations [14,15]. However, to date, no experimental evidence has been provided for its beneficial effects against diabetes-induced infertility, reproductive dysfunction, and inflammation. On these bases, in this study, we aimed to evaluate the effects of standardized hydroalcoholic extracts from the aerial part of *Portulaca oleracea* on streptozotocin-induced type-I-diabetes-associated reproductive system dysfunction and inflammation. 

## 2. Results

**Figure 1:**
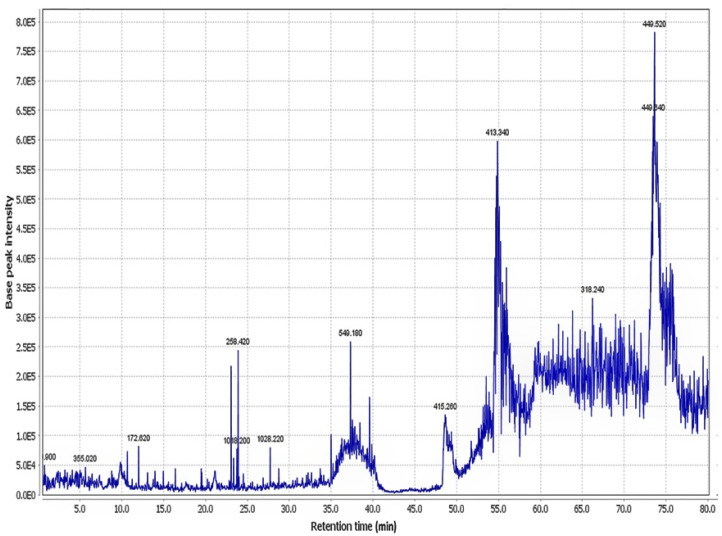
The total ion chromatogram of the aerial part of hydroethanolic extract of *Portulaca oleracea*. Reprinted from Ref. [12].

Collectively, 30 chemicals were characterized in the aerial parts of the hydroethanolic extract of PO, substantially including alkaloids (dopa; noradrenalin; and oleraceins A, B, C, and D), flavonoids, terpenoids (portulosides A and portulene), and vitamins (ascorbic acid, α-tocopherol, and riboflavin). Identified compounds are represented in Table 1. The total ion chromatogram of PO extract is also illustrated in Figure 1. 

### 2.1. The Effects of PO Extract on the Water and Food Consumption of Diabetic Rats

**Figure 2:**
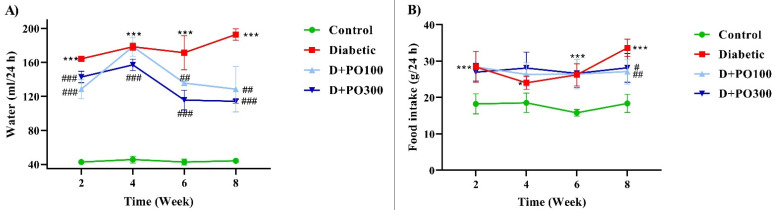
The effects of PO extract on (**A**) water and (**B**) food consumption in diabetic rats. The data are presented as mean ± SD. Repeated measures two-way ANOVA test was carried out with the following Tukey–Kramer’s post hoc test; * *p* < 0.05, *** *p* < 0.001 vs. control group; ^#^ *p* < 0.05, ^##^ *p* < 0.01, ^###^ *p* < 0.001 vs. diabetic group.

The food and water consumption were markedly elevated in the diabetic group compared to the control group at the end of two, four, six, and eight weeks of treatment (*p* < 0.05 and *p* < 0.001; Figure 2A,B). Furthermore, the PO extract (100 and 300 mg/kg) notably decreased the water consumption compared to the diabetic group at all experimental time-points (*p* < 0.01 and *p* < 0.001; Figure 2A). However, the food consumption was only reduced at the end of the eight weeks of treatment with PO extract (100 and 300 mg/kg) compared to the diabetic group (*p* < 0.05 and *p* < 0.01, respectively; Figure 2B).

### 2.2. The Effect of PO Extract on Blood Glucose Level

**Figure 3:**
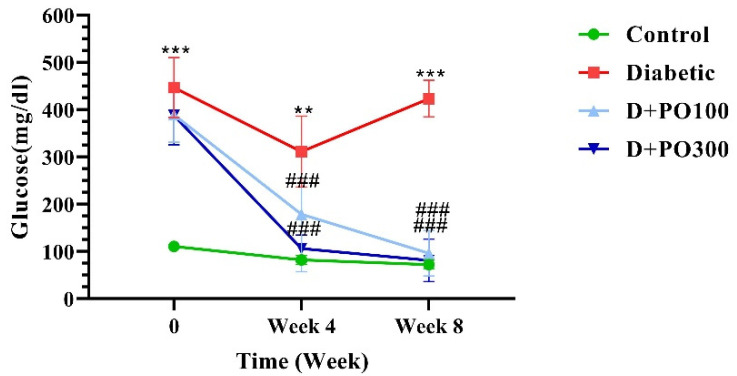
The effects of PO extract on blood glucose levels in diabetic rats. The data are presented as mean ± SD. Repeated measures two-way ANOVA test was carried out with the following Tukey–Kramer’s post hoc test; ** *p* < 0.01, *** *p* < 0.001 vs. control group; ^###^ *p* < 0.001 vs. diabetic group.

As reported in Figure 3, the blood glucose level was markedly enhanced in the diabetic group compared to the control group at the end of the zero, four, and eight weeks of treatment (*p* < 0.01 and *p* < 0.001; Figure 3). At the beginning of the study, there were no significant differences in the blood glucose levels of PO extract (100 and 300 mg/kg) and the diabetic group. However, following the four and eight weeks of treatment, PO extract (100 and 300 mg/kg) meaningfully diminished the blood glucose level compared to the diabetic group (*p* < 0.001 for all cases; Figure 3).

### 2.3. The Effect of PO Extract on Body Weight

**Figure 4:**
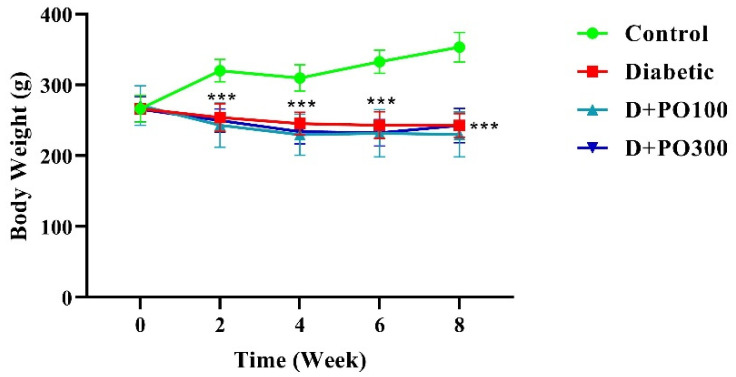
The effects of PO extract on body weight in diabetic rats. The data are presented as mean ± SD. A repeated-measures two-way ANOVA test was carried out with the following Tukey–Kramer’s post hoc test; *** *p* < 0.001 vs. control group.

At the beginning of the study, no significant differences were observed in the body weight between the control, diabetic, and PO extract groups (100 and 300 mg/kg; Figure 4). However, the diabetic group remarkably mitigated the body weight compared to the control group at the end of the two, four, six, and eight weeks of treatment (*p* < 0.001 for all cases, Figure 4). In addition, treatment with PO extract (100 and 300 mg/kg) could not significantly change the body weight compared to the diabetic group at all experimental time-points (Figure 4).

### 2.4. The Effect of PO Extract on the Testicular Weight and Testicular/Body Weight Index

**Figure 5:**
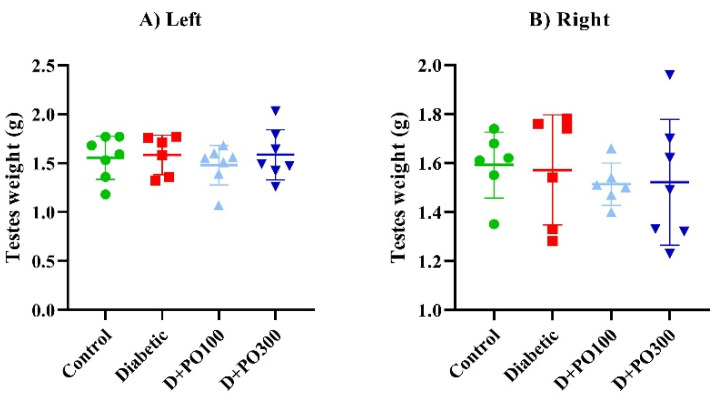

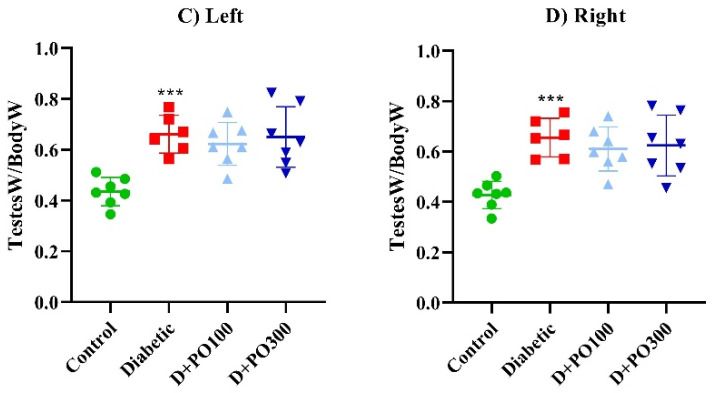
The effects of PO extract on (**A**) left and (**B**) right testicular weight and (**C**) left and (**D**) right testicular/body weight in diabetic rats. The data are presented as mean ± SD. A one-way ANOVA test was carried out with the following Tukey–Kramer’s post hoc test; *** *p* < 0.001 vs. control group.

At the end of the eight-week treatment, no significant differences were observed in the right and left testicular weight between the control, diabetic, and PO extract (100 and 300 mg/kg) groups (Figure 5A,B). Moreover, both the right and left testicular/body weight indexes were considerably augmented in the diabetic group more than the control group following the eight weeks of treatment (*p* < 0.001 for both cases; Figure 5C,D). However, eight weeks of treatment with PO extract (100 and 300 mg/kg) could not prevent the testicular/body weight index compared to the diabetic group (Figure 5C,D). 

### 2.5. The Effect of PO Extract on the Epididymis Weight and Epididymis/Body Weight

**Figure 6:**
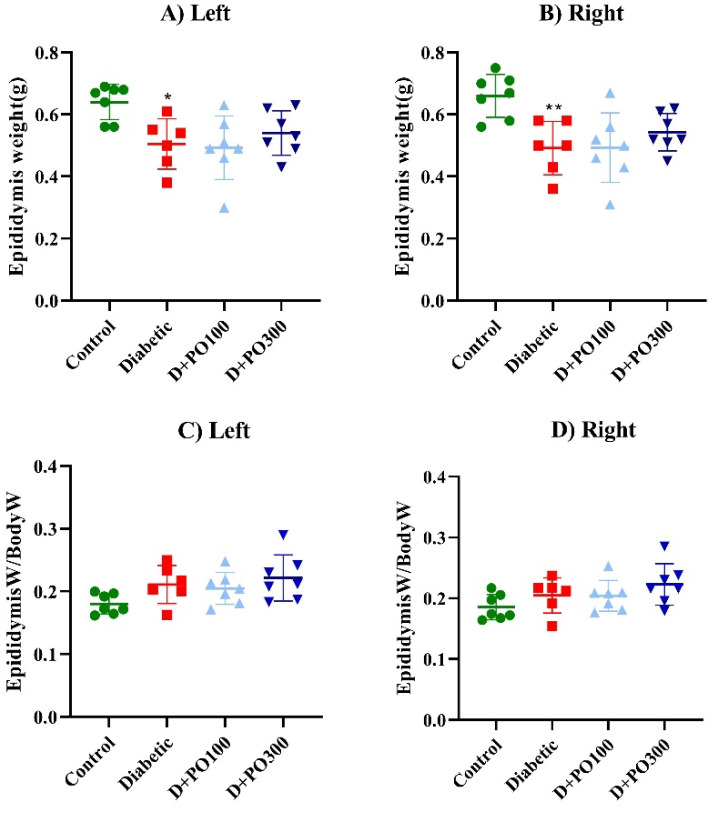
The effects of PO extract on (**A**) left and (**B**) right epididymis weight and (**C**) left and (**D**) right epididymis/body weight in diabetic rats. The data are presented as mean ± SD. A one-way ANOVA test was carried out with the following Tukey–Kramer’s post hoc test; * *p* < 0.05, ** *p* < 0.01 vs. control group.

As shown in Figure 6, the left and right epididymis weight was strikingly attenuated in the diabetic group compared to the control group at the end of the eight weeks (*p* < 0.05 and 0.01, respectively; Figure 6A,B). However, the PO extract (100 and 300 mg/kg) could not significantly change the left and right epididymis weight of the diabetic group following eight weeks of treatment (Figure 6A,B). Additionally, no significant differences were observed in the right and left epididymis/body weight index between the control, diabetic, and PO-extract (100 and 300 mg/kg) groups at the end of the eight weeks (Figure 6A,B). 

### 2.6. The Histopathological Evaluations

**Figure 7:**
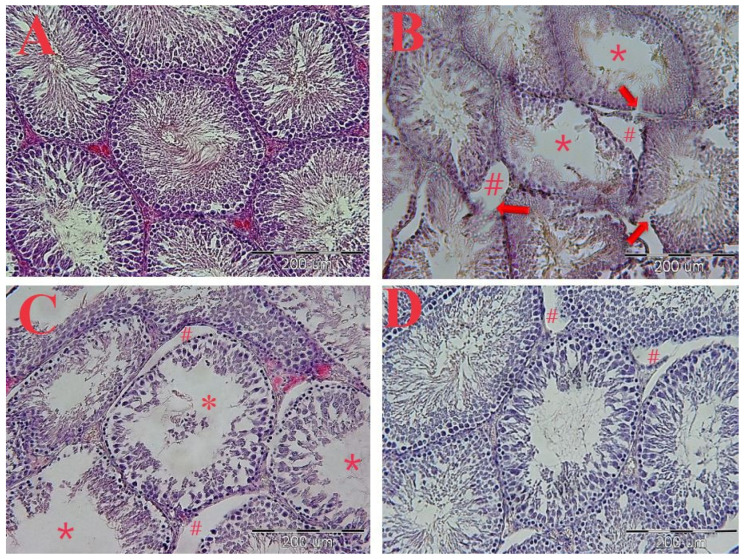
The effects of PO extract on the H&E staining of the transverse section of testicular tissue. (**A**) Control, (**B**) diabetic, (**C**) PO extract (100 mg/kg), and (**D**) PO extract (300 mg/kg). Microscopic view with magnification (20×) of the cross-section of testicular tissue. (**A**) Sham group: spermatogenic tubes with normal structural and cellular order. (**B**) Control group: geometric deformation of tubules, disintegration and rupture of the epithelium of spermatogenic tubules (arrows), reduction of spermatozoid population (*), increase in the distance between tubules, and atrophy and destruction of interstitial cells (Leydig) (#). (**C**) Treatment group with a dose of 100 mg/kg: The decrease in the density of spermatogenic cells is obvious, and there is a decrease in the number of sperm cells (*) and an increase in the distance between tubules (#). (**D**) treatment group with a dose of 300 mg/kg: a slight increase in the distance between tubules.

The results of the H&E staining showed the epithelium disintegration of seminiferous tubules, destruction of Leydig cells, increased space between the seminiferous tubules, and irregularities in the structure of the seminiferous tubules in the diabetic group compared to the control group (Figure 7A–D). However, the PO extract (100 and 300 mg/kg) firmly improved these changes compared to the diabetic group (Figure 7A–D). 

### 2.7. The Effect of PO Extract on the Diameter of the Seminiferous Tubules

**Figure 8:**
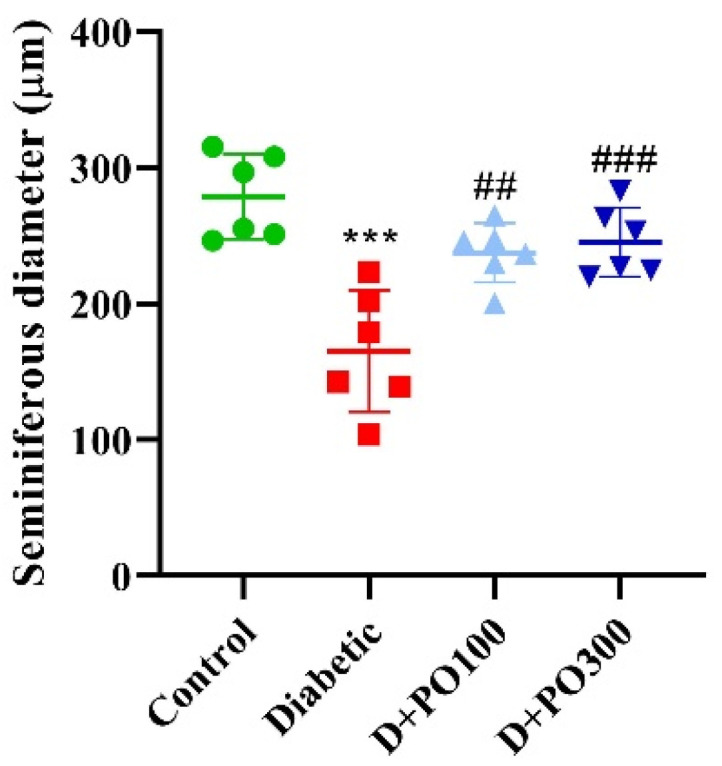
The effects of PO extract on seminiferous diameter in diabetic rats. The data are presented as mean ± SD. A one-way ANOVA test was carried out with the following Tukey–Kramer’s post hoc test; *** *p* < 0.001 vs. control group; ^##^ *p* < 0.01, ^###^ *p* < 0.001 vs. diabetic group.

Our results revealed that the diameter of the seminiferous tubules was meaningfully alleviated in the diabetic group compared to the control group (*p* < 0.001, Figure 8). Moreover, eight weeks of treatment with PO extract (100 and 300 mg/kg) provided a significant increment in the diameter of the seminiferous tubules compared to the diabetic group (*p* < 0.01 and 0.001, respectively, Figure 8).

### 2.8. The Effect of PO Extract on the Count and Motility of Sperm

**Figure 9:**
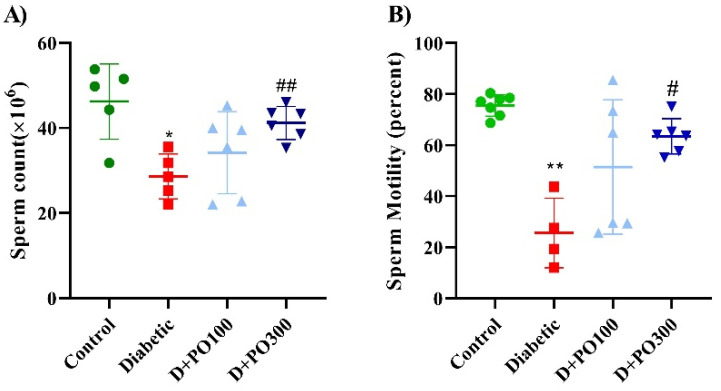
The effects of PO extract on (**A**) numbers and (**B**) motility of sperm in diabetic rats. The data are presented as mean ± SD. A one-way ANOVA test was carried out with the following Tukey–Kramer’s post hoc test; * *p* < 0.05, ** *p* < 0.01 vs. control group; ^#^ *p* < 0.05, ^##^ *p* < 0.01 vs. diabetic group.

At the experimental endpoint (eight weeks), the count and motility of sperm were considerably hampered compared to the control group (*p* < 0.05 and 0.01, respectively; Figure 9A,B). However, only the higher dose of PO extract (300 mg/kg) firmly propagated the count and motility of sperm compared to the diabetic group (*p* < 0.01 and 0.05, respectively; Figure 9A,B). 

### 2.9. The Effect of PO Extract on the Levels of LH, FSH, and Testosterone

**Figure 10:**
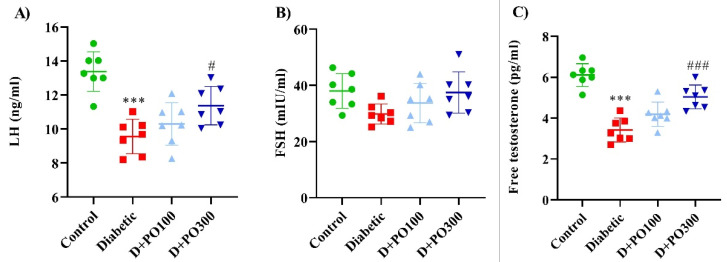
The effects of PO extract on (**A**) LH, (**B**) FSH, and (**C**) testosterone levels in diabetic rats. The data are presented as mean ± SD. A one-way ANOVA test was carried out with the following Tukey–Kramer’s post hoc test; *** *p* < 0.001 vs. control group; ^#^ *p* < 0.05, ^###^ *p* < 0.001 vs. diabetic group.

Our results showed that the diabetic group decreased the LH and testosterone levels significantly compared to the control group (*p* < 0.001 for both cases; Figure 10A,C). Reciprocally, eight weeks of treatment with PO extract (300 mg/kg) strikingly promoted the LH and testosterone levels compared to the diabetic groups (*p* < 0.05 and 0.001, respectively; Figure 10A,C). Furthermore, no significant changes in the FSH level were observed between the four studied groups at the experimental endpoint (eight weeks) (Figure 10C). 

### 2.10. The Effect of PO Extract on the Oxidative and Antioxidative Factors

**Figure 11:**
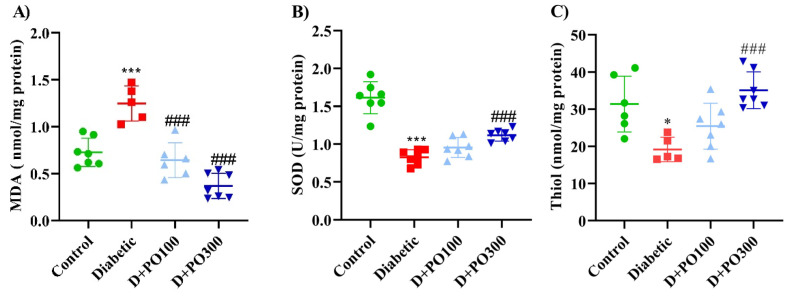
The effects of PO extract on (**A**) MDA, (**B**) SOD, and (**C**) thiol levels in diabetic rats. The data are presented as mean ± SD. A one-way ANOVA test was carried out with the following Tukey–Kramer’s post hoc test; * *p* < 0.05, *** *p* < 0.001 vs. control group; ^###^ *p* < 0.001 vs. diabetic group.

At the end of the eight weeks, the diabetic group notably elevated the MDA level compared to the control group (*p* < 0.001; Figure 11A), while the PO extract (100 and 300 mg/kg) markedly reduced the MDA level compared to the diabetic group (*p* < 0.001 for both cases, Figure 11A). Our results also revealed that the diabetic group significantly diminished the SOD activity and total thiol content compared to the control group (*p* < 0.001 and 0.05, respectively; Figure 11B,C). However, only the higher dose of PO extract (300 mg/kg) meaningfully enhanced the SOD activity and total thiol content compared to the diabetic group (*p* < 0.001 for both cases; Figure 11B,C).

### 2.11. The Effect of PO Extract on TNF-α, VEGF, and TGF-β Levels

**Figure 12:**
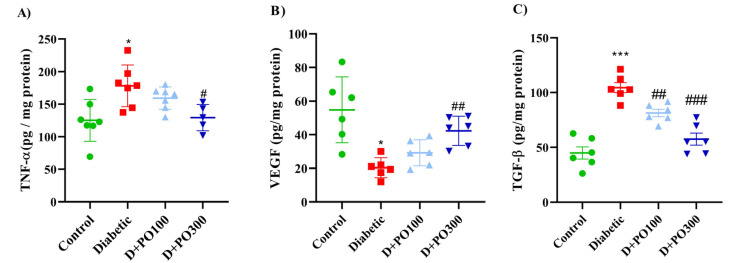
The effects of PO extract on the levels of (**A**) TNF-α, (**B**) VEGF, and (**C**) TGF-ꞵ levels in diabetic rats. The data are presented as mean ± SD. A one-way ANOVA test was carried out with the following Tukey–Kramer’s post hoc test; * *p* < 0.05, *** *p* < 0.001 vs. control group; ^#^ *p* < 0.05, ^##^ *p* < 0.01, ^###^ *p* < 0.001 vs. diabetic group.

As reported in Figure 12A, the TNF-α level was increased in the diabetic group compared to the control group (*p* < 0.05; Figure 12A), while considerably mitigated in the PO extract (300 mg/kg) group compared to the diabetic group (*p* < 0.05; Figure 12A). Our results also demonstrated that the diabetic group considerably attenuated the VEGF level compared to the control group (*p* < 0.05; Figure 12B). However, eight weeks of treatment with PO extract (300 mg/kg) notably propagated the VEGF level compared to the diabetic group (*p* < 0.01, Figure 12B). On the contrary, the TGF-β level was remarkably increased in the diabetic group compared to the control group (*p* < 0.001; Figure 12C), while the PO extracts (100 and 300 mg/kg) considerably mitigated the TGF-β level compared to the diabetic group (*p* < 0.01 and *p* < 0.001, respectively; Figure 12C).

## 3. Discussion

Our study, for the first time, demonstrates the protective effect of *Portulaca oleracea* on streptozotocin-induced type-I-diabetes-associated reproductive system dysfunction and inflammation. Several studies, in both diabetic animal models and on human cavernosal tissue from diabetic patients, have demonstrated that the erectile dysfunction associated with diabetes is a multifactorial condition involving inflammation, oxidative damage and metabolic disorders [31]. Accordingly, our results showed that streptozotocin-induced diabetes leads to increased oxidative stress and inflammatory markers in the testicular tissue and impaired fertility parameters and that PO extract strongly ameliorated these parameters. This seems to be related to the presence of oleraceins (A, B, C, and D), flavonoids, terpenoids (portulosides A and portulene), and vitamins (ascorbic acid, α-tocopherol and riboflavin) chemically characterized for the plant extract.

Pieces of evidence have proved the induction of diabetes and hyperglycemia through a single i.p. injection of streptozotocin in rats [32]. Additionally, Akbarzadeh and co-workers supported that the blood glucose level, food and water consumption, and urine volume were markedly increased, while the body weight and insulin level were reduced, following streptozotocin-induced diabetes in rats [33]. Consistently, streptozotocin-induced diabetes augmented the blood glucose level and food and water consumption, while strikingly decreasing body weight [34]. Our results showed that the streptozotocin-induced diabetes group notably enhanced the blood glucose level and food and water consumption, while significantly reducing the body weight compared to the control group. In addition, PO extract (at both 100 and 300 mg/kg) significantly decreased the blood glucose level and food and water consumption without affecting rat body weight. 

According to our results, Lee et al. demonstrated that aqueous extract of PO (300 mg/kg; oral gavage for ten weeks) diminished the blood glucose, triglyceride, low-density lipoprotein (LDL)-cholesterol levels, while elevating the insulin and high-density lipoprotein (HDL)-cholesterol levels in diabetic db/db mice [35] and alloxan-induced diabetic rats [36,37]. Notably, the present study also demonstrates that streptozotocin-induced diabetes leads to the epithelium disintegration of seminiferous tubules, the destruction of Leydig cells, and increased space between the seminiferous tubules, and irregularities in the structure of the seminiferous tubules in the testicular tissue. Moreover, the seminiferous tubules’ diameter, sperm count, and sperm motility were diminished in the diabetic group. In line with our results, Ricci and co-workers showed the abnormal histology, seminiferous epithelium cytoarchitecture, occludin distribution pattern, hypertrophy, and abnormally distribution of Leydig cells in the testicular tissue of the streptozotocin-induced diabetes rats. They also reported a decrease in the testosterone and SOD levels in diabetic rats [38]. Consistently, streptozotocin-induced diabetes leads to desquamation of spermatids in the lumen and disorganization of seminiferous tubule germinal epithelium in the testicular tissue.

Furthermore, the LH and testosterone levels and the mean seminiferous tubule diameter were diminished in the diabetic rats [39]. In addition, recent studies reported a decrease in mean seminiferous tubule diameter and sperm count and motility following streptozotocin administration [40,41,42]. Our results show that PO extract remarkably improved the histopathological changes in the testicular tissue and increased the seminiferous tubule diameter; these results are in accordance with previous reports that demonstrated a protective effect of PO extracts in sperm count and testosterone level in male albino rats [40,43,44,45]. 

The male reproductive system is regulated through the hypothalamic–pituitary–testicular axis. The gonadotropin-releasing hormone (GnRH) is secreted by the hypothalamus and stimulates the secretion of LH and FSH from the pituitary gland [46]. As a consequence, LH stimulates the testosterone secretion through Leydig cells, while the FSH regulates spermatogenesis by affecting the Sertoli cells of the testes [47] and testosterone levels [48]. This evidence indicates that there is a relationship between insulin/glucose and LH/FSH levels in serum and that their ratio is affected in diabetes. However, the mechanisms by which insulin, glucose, or both control these hormones are unclear [49].

In our study, we found that the LH and testosterone levels were mitigated, while the FSH level did not change in the streptozotocin-induced diabetes group. Additionally, we found that the higher dose of PO extract (300 mg/kg) firmly propagated the LH and testosterone levels while not changing the FSH level, following streptozotocin-induced diabetes. Similarly, Farag et al. demonstrated that PO seeds’ extract (200 and 400 mg/kg) prevented testicular dysfunction, while enhancing testosterone levels following the acrylamide-induced testicular toxicity in rats [50]. In another study, PO seeds and shoot extract (50 mg/kg) significantly increased LH, FSH, and testosterone levels following the doxorubicin-induced testicular toxicity in albino rats [51].

Recent pieces of evidence emphasized the relation between oxidative stress and male infertility. Indeed, DM-1 can affect the spermatogenesis by oxidative damage generating reactive oxygen species (ROS), which either affect the cellular antioxidant defense mechanisms or directly stimulate the inflammatory signaling pathways, ending in testicular apoptosis [52]. Thus, ROS attenuation is crucial for the treatment of reproductive damage in diabetic patients. Additionally, numerous studies emphasized that streptozotocin-induced diabetes elevated lipid peroxidation and ROS levels, while mitigating anti-oxidative markers, including catalase, SOD, glutathione peroxidase, glutathione transferase, and glutathione reductase activities in the testis and epididymal sperm [53,54]. In our preclinical assessment, we showed that streptozotocin-induced diabetes elevated the MDA level, while also attenuating the total thiol content and the SOD activity in the testicular tissue. Moreover, PO extract notably ameliorated the oxidative stress induced by streptozotocin in the testicular tissue of rats. This is in line with previous works that demonstrated the anti-oxidative properties of PO in both male [55,56] and female rodents [57,58]. 

The last point that we want to discuss is the pivotal role of inflammation in diabetic testicular complications [59] and the stringent involvement of VEGF (an angiotrophic and neurotrophic factor) [60] on spermatogenesis and Sertoli and Leydig cells’ physio-pathology. The importance of interferon-gamma (IFN-γ), interleukin (IL)-1β, and TNF-α in these physio-pathological mechanisms has been well clarified [61,62,63]. Furthermore, it has also been revealed that increased TNF-α (in semen) and IL-1β, and IL-6 are associated with decreased sperm count, motility, and morphology [64]. upon the onset and during the progression of diabetes [65,66]. Additionally, it has been reported that VEGF supports germ cell proliferation and survival and regulates endothelial permeability and microcirculation in the testis [67,68]. Moreover, different report highlight that streptozotocin-induced diabetes mitigated the VEGF level, associated with increased apoptosis and testicular damage in rats [69,70,71]. In our investigation, we found that PO was able to increase the levels of VEGF and to revert the streptozotocin-induced increased of TNF-α and TGF-β levels in testicular tissue. Taken together, these results further corroborate the protective effects of PO on type-I-diabetes-associated reproductive system dysfunction and inflammation.

## 4. Materials and Methods

### 4.1. Drugs and Chemicals

Streptozotocin, dimethyl sulfoxide (DMSO), and ethanol were prepared from Sigma-Aldrich Chemical Co. (St. Louis, MO, USA). Ketamine and xylazine were obtained from ChemiDaru Company (Tehran, Iran). Tumor necrosis factor-alpha (TNF-α) and vascular endothelial growth factor (VEGF) and transforming growth factor-beta (TGF-β) ELISA kits were purchased from IBL-International^®^ Company (Hamburg, Germany), and luteinizing hormone (LH), follicle-stimulating hormone (FSH), and testosterone ELISA kits were prepared from CUSABIO Company (Eco-Life Science Ltd., Hong Kong, China). Furthermore, malondialdehyde (MDA), superoxide dismutase (SOD), and total thiol content kits were prepared by Zell Bio Company (Lonsee, Baden-Württemberg, Germany). Other chemicals or reagents were also provided at analytical grades from Santa Cruz Biotechnology (Santa Cruz, Dallas, TX, USA).

### 4.2. Preparation of Portulaca oleracea Extract and Liquid Chromatography-Mass Spectrometers (LC-MS) Characterisation

*Portulaca oleracea* (PO) was collected from Sabzevar, Khorasan Razavi province, Iran, in July 2020 and was identified by the pharmacy school at Mashhad University of Medical Sciences (herbarium No. 12-1615-240). First, the extract was prepared by using the maceration method described previously. In brief, 100 g of aerial parts of PO was soaked with one lit 70% ethanol for 48 h at room temperature. Then the extract was concentrated with a rotary evaporator and freeze-dried [7,8,9,10,11]. The yield of the dried extract was 19.5% *w*/*w* and stored at −20 °C until use. Finally, the PO concentrations (100 and 300 mg/kg) were prepared with sterilized distilled water containing 1% *v*/*v* DMSO from the raw extract. The liquid chromatography–mass spectrometers (LC–MS) characterization was performed by an AB SCIEX QTRAP (Shimadzu) liquid chromatography coupled with a triple quadrupole Mass Spectrometer and using a Supelco C18 (15 mm × 2.1 mm × 3 μm) column. It was performed according to our previously published methods [12]. The mass spectra were obtained by scanning time of 80 min and in a range of 100 to 1700. The mass spectra were obtained by a scanning time of 80 min and in a range of 100 to 1700. The positive electrospray ionization (ESI) mode was applied for the Mass Spectrometer. 

### 4.3. Animals’ Husbandry and Ethics

In this study, 28 healthy male Wistar rats weighing 250–300 g were obtained from the animal care center, Mashhad University of Medical Sciences. The rats were deployed in separated standard cages and ventilated rooms with a 12/12 h natural light–dark cycle, temperature of 24 ± 2 °C, and humidity of 60 ± 3%, with food and water ad libitum. All animals received human care in compliance with Mashhad University of Medical Sciences guidelines (Ethical Approval Code, 980077; Approval Date, 8 August 2019; Approval ID, IR.MUMS.MEDICAL.REC.1398.563). Animal procedures were performed according to ARRIVE guidelines and the Basel declaration, including the 3Rs concept. All methods were carried out to minimize the number of animals used (*n*= 7 per group) and their suffering. Experimental study groups were randomized, and their assessments were carried out by researchers blinded to the treatment groups.

### 4.4. Experimental Diabetes Induction

Diabetes was induced by a single intraperitoneal (i.p.) injection of streptozotocin (50 mg/kg). The streptozotocin solution was freshly prepared in a cold 0.1 M citrate buffer (pH = 4.5). The serum glucose level was measured by using a glucometer after 72 h of streptozotocin injection to confirm the diabetes model. The animals with fasting glucose levels higher than 220 mg/dL and signs of polyuria and polydipsia were considered diabetic [34].

### 4.5. Study Design

Twenty-eight healthy male Wistar rats (250–300 g) with proven fertility were randomly divided into four experimental groups, as described below:

1. Control group: receiving a single dose of streptozotocin carrier (with a volume equal to 50 mg/kg; i.p.) + PO extract vehicle (oral gavage; for eight weeks).
2. Diabetic group: receiving a single dose of streptozotocin (50 mg/kg; i.p.) + PO extract vehicle (daily oral gavage; for eight weeks).
3. Treatment group-1: receiving a single dose of streptozotocin (50 mg/kg; i.p.) + PO extract (100 mg/kg; daily oral gavage for eight weeks).
4. Treatment group-2: receiving a single dose of streptozotocin (50 mg/kg; i.p.) + PO extract (300 mg/kg; daily oral gavage for eight weeks).

The selected dose was chosen based on preliminary experiments and previous works [8,15,50,72].

### 4.6. Measurement of Blood Glucose Levels 

The 12 h fasting blood glucose level was measured with Accu-Chek Active^®^ glucometer (Roche Diagnostics GmbH, Mannheim, Germany), using the tail vein, at the end of the zero, four, and eight weeks of treatment.

### 4.7. Sample Preparation

At the experimental endpoint, rats were deeply anesthetized with ketamine (100 mg/kg) and xylazine (10 mg/kg) and acepromazine (3 mg/kg) [12,73]. The blood samples (2 cc) were gathered by intracardiac puncture and then straightly centrifuged at 3000 rpm for 10 min at 4 °C, and the supernatants (sera) were isolated and kept at −20 °C for further investigations. The testicular and epididymis were sequestered and weighed. The testicular tissue homogenate (10% *w*/*v*) was provided in 5% potassium chloride and 0.5 mM PMSF, and the protease inhibitor cocktail was then centrifuged at 3000 rpm for 10 min, at 4 °C. The supernatants were collected and stored at −20 °C for further investigations. According to Bradford’s method, the total protein concentrations were measured [12,74]. In addition, the epididymis was fixed in 10% *v*/*v* buffer formalin for histopathological assessments and was soaked with saline to evaluate the number and motility of sperm. 

### 4.8. Assessment of Hormonal Factors

Enzyme-linked immunosorbent assays (ELISA) for LH, FSH, and testosterone levels were carried out on serum samples according to the manufacturer’s instructions [75]. 

### 4.9. Assessment of Oxidative and Anti-Oxidative, Inflammatory, Fibrosis and Angiogenesis Biomarkers

The levels of MDA, as an oxidative factor, and the total thiol content and the SOD activity, as anti-oxidative factors, were determined in the testicular tissue by using commercial biochemistry kits according to the manufacturer’s instruction [76]. In addition, the levels of TNF-α, as an index of inflammation; VEGF levels, as an angiogenesis marker; and TGF-β, as a fibrotic factor, were measured in testicular tissue by using ELISA assay [7,10,11,12,77].

### 4.10. Histological Evaluations and Measurement of the Seminiferous Diameter, Sperm Count and Motility

After sacrifice, the animals’ abdomens were opened to weigh and evaluate the fertility parameters, including testicular weight, relative testicular weight, epididymal weight, motility and number of sperm, and protein-level measurement. After the dissection of the lower region, the tail of the epididymis was removed and transferred to a container containing physiological serum. After dissecting the tail and removing the sperm, the remaining tissue fragments were separated from the suspension. The resulting sperm suspension was counted at a ratio of 1:20 to calculate the number of sperm under a light microscope, using a NeoBar slide. The testicular tissue was fixed with 10% *v*/*v* buffered formalin, and the histological process, including dehydrating, clearing, and embedding, was carried out. After that, the microscopic sections (5 μm) were prepared and stained with Hematoxylin and Eosin (H&E) and evaluated by optical microscopy. The average seminiferous diameter (μm) was determined for each testis [78]. 

### 4.11. Statistical Analysis

All collected data were analyzed by using Graph Pad Prism^®^ 8 (Graph Pad Software, San Diego, CA, USA) software and expressed as mean ± SD. Initially, the normality of the data distribution was evaluated by using the Kolmogorov–Simonov test. In the next step, the biochemical and oxidative result comparison was carried out using a one-way analysis of variance (ANOVA) with Tukey–Kramer’s post hoc test. In addition, a comparison of the results of blood glucose levels, weight, and food and water consumption was made by using the repeated measures two-way ANOVA test with Tukey–Kramer’s post hoc test. The probability (P) values were considered statistically significant when *p* ≤ 0.05, 0.01, and 0.001. By data normalization, animal weight was used for randomization and group allocation to reduce unwanted sources of variations. No animals and related ex vivo samples were excluded from the analysis. An in vivo study was carried out to generate groups of equal size (*n* = 7 of independent values), using a randomization and blinded analysis.

## 5. Conclusions

The results of this study demonstrate, for the first time, the protective effect of *Portulaca oleracea* (phytochemically characterized by using liquid chromatography–mass spectrometry to detect bioactive molecules) on streptozotocin-induced type I diabetes–associated reproductive system dysfunction and inflammation. These effects are most likely attributable to (i) the decreasing of blood glucose level and testicular tissue damage, (ii) the improvement of inflammatory factors, and (iii) the modulation of sex hormones level and fertility potential. Therefore, these findings indicate the promising beneficial role of PO extract as an efficient therapeutic agent for treating diabetic infertility. However, further preclinical studies aimed to identify the mechanism/s of action, and, potentially, clinical trials are necessary to support and corroborate this evidence.

## Figures and Tables

**Table 1:** The peak of chemicals in the hydroethanol extract of *Portulaca oleracea*, using positive mode LC–MS.

Peak No.	Compound Identification	t_R_ (min)	M+H (*m*/*z*)	Reference
1	Portulacanone D	26.9	299.76	[16]
2	Noradrenaline	37.0	170.7	[17]
3	Dopa	15.0	198.12	[18]
4	Oleraceins A	62.5	504.66	[18]
5	Oleraceins B	9.5	533.76	[18]
6	Oleraceins C	64.1	666.06	[18]
7	Oleraceins D	13.1	696.84	[18]
8	Adenosine	19.8	268.8	[18]
9	(3R)-3,5-Bis(3-methoxy-4-hydroxyphenyl)-2,3-dihydro-2(1H)-pyridinone	89.3	342.36	[19]
10	Aurantiamide acetate	36.4	445.8	[20]
11	Cyclo(L-tyrosinyl-L-tyrosinyl)	67.7	327.24	[20]
12	Portuloside A	72.2	332.22	[21]
13	Portulene	66.3	337.02	[22]
14	Lupeol	66.5	427.5	[22]
15	(3S)-3-O-(β-D-Glucopyranosyl)-3,7-dimethylocta-1,6-dien-3-ol	67.8	318.12	[23]
16	Friedelane	54.9	413.34	[24]
17	Quercetin	39.4	303.18	[25]
18	Myricetin	55.1	318.24	[25]
19	Genistin	65.4	433.20	[26]
20	Indole-3-carboxylic acid	77.8	162.90	[16]
21	Palmitic acid	62.2	256.14	[27]
22	Stearic acid	37.8	285.18	[27]
23	Caffeic acid	65.8	181.08	[28]
24	Riboflavin	35.0	376.62	[29]
25	Vitamin C	28.5	177.00	[29]
26	α-Tocopherol	67.1	431.22	[27]
27	Hesperidin	76.8	611.58	[30]
28	Portulacerebroside A	64.6	843.18	[24]
29	β-Sitosterol	48.7	415.32	[22]
30	β-Carotene	37.5	538.74	[27]

## Data Availability

The datasets generated and/or analyzed during the current study are available from the corresponding author upon reasonable request.

## Sample Availability Statement

Samples of the compounds are available from the authors.
