# Correction: Rakhshandeh et al. Protective Effect of *Portulaca oleracea* on Streptozotocin-Induced Type I Diabetes-Associated Reproductive System Dysfunction and Inflammation. *Molecules* 2022, *27*, 6075

**DOI:** 10.3390/molecules30030449

**Published:** 2025-01-21

**Authors:** Hassan Rakhshandeh, Hamed Rajabi Khasevan, Anella Saviano, Mohammad Reza Mahdinezhad, Vafa Baradaran Rahimi, Sajjad Ehtiati, Leila Etemad, Alireza Ebrahimzadeh-bideskan, Francesco Maione, Vahid Reza Askari

**Affiliations:** 1 Pharmacological Research Center of Medicinal Plants, Mashhad University of Medical Sciences, Mashhad 9177948564, Iran; rakhshandehh@mums.ac.ir (H.R.);; 2 ImmunoPharmaLab, Department of Pharmacy, School of Medicine and Surgery, University of Naples Federico II, Via Domenico Montesano 49, 80131 Naples, Italy; anella.saviano@unina.it; 3 Department of Clinical Biochemistry, Faculty of Medicine, Mashhad University of Medical Sciences, Mashhad 9177948564, Iran; 4 Department of Cardiovascular Diseases, Faculty of Medicine, Mashhad University of Medical Sciences, Mashhad 9177948564, Iran; baradaranrv@mums.ac.ir; 5 Pharmaceutical Research Center, Pharmaceutical Technology Institute, Mashhad University of Medical Sciences, Mashhad 9177948564, Iran; etemadl@mums.ac.ir; 6 Department of Drug Control, School of Pharmacy, Mashhad University of Medical Sciences, Mashhad 9177948564, Iran; 7 Department of Anatomy and Cell Biology, Faculty of Medicine, Mashhad University of Medical Sciences, Pardis Campus, Azadi Square, Mashhad 9177948564, Iran; ebrahimzadehba@mums.ac.ir; 8 Applied Biomedical Research Center, Mashhad University of Medical Sciences, Mashhad 9177948564, Iran; 9 Department of Pharmaceutical Sciences in Persian Medicine, School of Persian and Complementary Medicine, Mashhad University of Medical Sciences, Mashhad 9177948564, Iran; 10 International UNESCO Center for Health-Related Basic Sciences and Human Nutrition, Mashhad University of Medical Sciences, Mashhad 9177948564, Iran

In the original publication [1], there was a mistake in the caption of Figure 1 as published. The authors unintentionally reused chromatograms from previous publications but neglected to properly cite the previous work. However, the authors referenced the previous publication as Reference [12] in the main text of the original publication [1]. The correct figure caption appears below. The authors state that the scientific conclusions are unaffected. This correction was approved by the Academic Editor. The original publication has also been updated.

**Figure 1.** The total ion chromatogram of the aerial part of hydroethanolic extract of *Portulaca oleracea.* Reprinted from Ref. [12].
